# Conceptions of university students on microplastics in Germany

**DOI:** 10.1371/journal.pone.0257734

**Published:** 2021-09-23

**Authors:** Patricia Raab, Franz X. Bogner

**Affiliations:** Department of Biology Education, Centre of Maths and Science Education (Z-MNU), University of Bayreuth, Bayreuth, Germany; Kaohsiung Medical University, TAIWAN

## Abstract

Microplastics are a global challenge and a frequently studied environmental issue. Hence, the knowledge body about microplastics within the scientific community is growing fast and challenges an elaborated knowledge transfer from science to the general public. Just as well-informed people are the basis for reducing microplastics’ impact on the environment, knowledge of the audience’s conceptions is the basis for an accurate and successful dissemination of scientific findings. However, insights into the publics’ perceptions of microplastics are still rare. The present study aimed to capture students’ conceptions about microplastics based on their individual experiences following qualitative inductive, exploratory research. Therefore, 267 students of a state university in Germany responded to a paper-and-pencil questionnaire containing open and closed questions on microplastic-related conceptual understanding, risk perception, information behavior, sources, and sinks. The inductive classifying of all responses by a qualitative content analysis revealed six basic concepts: 1) Microplastics are mainly understood as small plastic particles. 2) Microplastics are closely associated with its negative consequences. 3) The most labeled source in households is plastic packaging. 4) Compared to other water bodies, microplastics are rarely suspected in groundwater. 5) A high threat awareness exists in classifying microplastics as very dangerous and dangerous. 6) Media such as TV or the Internet are the most crucial information sources while the school has less importance in acquiring information. It is precisely this pattern that indicates the need for profound science communication to establish a joint and scientifically sound knowledge base in society. Knowledge about conceptions of potential “customers” allows tailor-made scientific knowledge transfers to shape public awareness, initiate changes in thoughts and prepare the field for collaborative behavior.

## Introduction

### Scientific background

The success of plastic materials transformed life and, at the same time, challenged our planet permanently and irrevocably. In 2017, Geyer et al. [1] estimate the number of plastics ever manufactured at 30%, which is still in use since the beginning of production in 1950. The rest (70%) is classified as waste and was either incinerated, recycled, or, representing the largest share, with 79%, accumulated in landfills or the environment [1]. Consequently, not surprisingly, plastic pollution is one of our generation’s critical environmental challenges [2]. In addition to the anesthetic and easily visible plastic debris, smaller plastic fragments, namely microplastics, contribute to worldwide environmental pollution. Microplastics are defined as plastic particles smaller than 5 mm [3] and are classified into primary and secondary microplastics [4]. Primary microplastics are intentionally manufactured in a micro-size range to find use in plastic production as plastic resin pellets, industrial abrasives, and cosmetic products [5]. Secondary microplastics are fragmented larger-sized plastic materials caused, for instance, due to exposure to UV light and mechanical forces [6]. However, microplastics are a very diverse group: they differ greatly according to their color, shape, size, chemical composition, and specific density [7, 8].

Duis & Coors [7] meta-analyzed primary and secondary microplastic source studies, compiling an overview of potential sources, which we allocated to the different sectors (see Table 1). Sources such as building materials [9] or sewage sludge in agricultural sites [10] are still missing. The chosen structure to apportion the sources in different sectors offers one possible classification: building materials, road paints, and automobile tire wear may likewise summarize the urban run-off [11].

**Table 1:** Listing of primary and secondary microplastic sources.

	Primary Microplastic Sources	Secondary Microplastic Sources
**Household**	Personal care products containing microplastics as exfoliants/abrasives	Abrasion/release of fibers from synthetic textiles Release of fibers from hygiene products Abrasion from other plastic materials (*e*.*g*., household plastics) Plastic items in organic waste
**Industry**	Drilling fluids for oil and gas exploration Industrial abrasives Pre-production plastics, production scrap, plastic regranulate: accidental losses, run-off from processing facilities	Paints based on synthetic polymers (ship paints, other protective paints, house paint, road paint): abrasion during use and paint removal, spills, illegal dumping Plastic coated or laminated paper: losses in paper recycling facilities
**Agriculture**		Plastic mulching Synthetic polymer particles used to improve soil quality and as composting additive
**Maritime Activity**		Material lost or discarded from fishing vessels and aquaculture facilities Material lost or discarded from merchant ships (including lost cargo), recreational boats, oil and gas platforms
**Traffic**		Abrasion from car tires
**Plastic Litter**		General littering, dumping of plastic waste Losses of waste during waste collection, from landfill sites and recycling facilities
**Medicines**	Medical applications (*e*.*g*., dentist tooth polish)	
**Weather**		Losses of plastic materials during natural disasters

Modified after Duis & Coors [7].

The respective share of primary and secondary microplastics to the total contamination of microplastics in the environment lacks reliable quantification [12]. A view widely held expects large plastics as a major source of microplastics in the environment, which consequently disintegrates into secondary microplastics through external influences.

Plastics inevitably enter the three environmental compartments water, atmosphere, and soil [9]. First reports on microplastics in the oceans date back to the 1970s [13]. In the sea, many studies reported microplastics from the surface [14, 15] to the sediments [14, 16] down to the deep sea [17]. Zbyszewski & Corcoran [18] documented microplastics in the Lake Huron in the US. Imhof et al. [19] did it for the Lake Garda in Europe. Expectedly, also the terrestrial system is charged with microplastics. Microplastics are present in cities in the form of tire abrasion [20, 21], agricultural sites, with fibers having the largest share [10], as well as in remote regions like the French Pyrenees [2], the arctic [22], and groundwater [23, 24]. Finally, atmospheric transport distributes microplastics worldwide, even in regions with no or sparse human population, by wet and dry deposition [2].

Regarding the consequences, Laist [25] estimated 267 marine species to be affected by plastic debris. Plastic ingestion was also reported for other marine animals such as turtles [26–28], fish [29], and marine birds [30, 31]. Next to plastics, also the already investigated effects of microplastics in the different ecosystems appear far-reaching. Microplastics’ small size raises its accessibility to a broader range of organisms [6, 32]. Many researchers reported the ingestion of microplastics by marine animals like zooplankton [33, 34] and mollusks [35] as well as by crabs [36, 37], fish [38, 39], birds [40], and whales [41]. Moreover, microplastics have also been detected in freshwater animals like water flea [42], fish [43, 44], and worms [45].

After ingestion, microplastics can translocate from the gut to the circulatory system and tissues [46], from tissues to cells [47], and organs [37]. Chemicals adsorbed on the surface of microplastics may accumulate in the body after ingestion and may have health implications (*e*.*g*., lead to liver toxicity and pathology in fish) [48]. After uptake, microplastics could be transferred to higher trophic levels [49]. Beginning with zooplankton, microplastics may accumulate in the marine food web [50], wherefore, it comes as no surprise to assume microplastics also in the human food chain [51]. Vinay Kumar et al. [52] detected microplastics in mussels sold in supermarkets for human consumption. Next to marine organisms, contamination of food during production or packaging is a possible microplastic source for human consumption, *e*.*g*., in bottled water [53] or salt [54].

Besides ingestion, further consequences start from the presence of microplastics in the environment. Organisms, *e*.*g*., bacteria, use microplastics for settlement and hitchhike through the waters [55]. Microplastics can change soil properties like their structure and water dynamics in the terrestrial ecosystem, thereby possibly affecting plant performance, *e*.*g*., its biomass or root traits [56]. Moreover, microplastics are in the air we breathe [57, 58], travel on the wind, and drift down the skies to remote regions [22].

### Students’ conceptions

Despite the growing research body, only a few social science studies address microplastics’ social perception [59]. Therefore, there is a need to understand which and how much scientific knowledge arrives in the general public. This may help offer target-oriented awareness campaigns on the responsible handling of plastic and microplastics. To generate insights into individual conceptions about microplastics, we chose university students who had successfully passed secondary school education. Consequently, their conceptions may provide crucial implications for science communication.

The application of qualitative content analysis allows the categorization of a great variety of individual conceptions. This method follows the theory of constructivism, which evolved from Piaget’s studies of cognitive development [60]. Every person holds particular conceptions about the world, which may differ from those others have and are used to orientate themselves in the world and explain natural phenomena. They are formed in the early years of life and are stable in the face of change [61]. In this regard, the demarcation of knowledge and conceptions is not entirely unambiguous. Basically, conceptions differ from knowledge in that they have no claim to truth and are more subjective [62]. For many years, research focused on the differences between scientific conceptions and the so-called misconceptions that education should replace. Today, everyday (naïve) students’ conceptions are labeled as alternative conceptions, a term introduced by Wandersee et al. [63], indicating their lifeworld reference separated from professional conceptions. Gathering insight into students’ conceptions shows their individual conceptions based on personal experience, often differing from scientific ones [64]. Already in 1994, Smith et al. ([65], p. 151) labeled prior knowledge as “primary resource for acquiring new knowledge.” Hence, conceptions represent good starting points for awareness campaigns to develop scientific comprehension [60]. Successful science education rests on, among other things, the consideration and inclusion of individual conceptions [66], which may slowly refine and transform. Therefore, communicational efforts, tailor-made to conceptions and experiences, may result in better outcomes.

Research lines on conceptions exist as a convenient tool for successful teaching in science classes [64]. Several studies uncovered diverse conceptions of university students [67] and pupils on scientific topics [68–70]. Fröhlich et al. [71] reported a change of perceptions during the school career. Following the constructivist view of learning, new knowledge is constructed by rearranging the existing cognitive basis through experience [65]. For enlightenment, initiatives must give students a chance to promote their effort in combining existing with new information [72]. Thereby, learning itself can be a very individual process [73]. Knowledge of students’ conceptions and incorporation of them into the process of learning may lead to more advanced and comprehensive learning [60], possibly overcoming alternative conceptions [74]. Franke & Bogner [75] applied alternative conceptions in their gene technology laboratory intervention. Divided into two treatment groups, one was confronted with alternative conceptions on the underlying topic during the lesson, while the other was not. The pupils who dealt with the alternative conceptions showed higher interest and well-being as well as a better cognitive achievement proving the relevance of conceptions’ recognition [75]. Finally, conceptions indicate neglected topics in the curriculum and give suggestions for an integration in the future [71].

As already mentioned, currently, little is known about the general public’s comprehension of microplastics. We have chosen a student sample representing the final stage of school education, giving insights into the conceptions students hold at the very end of their school careers. Thereby, university students provide school and university education implications in particular and science communication implications in general. Educators and scientists get an impression of topics surrounding microplastics that need to be deepened, put into a different context, or become part of the curriculum in the first place. Additionally, in the context of science communication, scientists may get an idea of the level of general understanding to communicate their knowledge at an appropriate level. Consequently, our study monitors different topics surrounding microplastics which give valuable starting points for a variety of awareness campaigns. The overall aim of the study was to receive first insights into the conceptions university students hold on microplastics to make science communication of any kind more precise and adequate. The study’s objectives were five-fold: (1) What do German university students understand by the term microplastics? (2) Where do students get their information about microplastics from? (3) Which microplastic sources in the household do students know? (4) Which water ecosystems in Germany do students consider contaminated with microplastics? (5) How dangerous do students consider microplastics, and how do they justify their classification?

## Materials and methods

In this study, we followed the exploratory research [76], aiming to provide meaningful first insights into students’ conceptions on microplastics in Germany. We decided on combining the exploratory research with a qualitative inductive research method [77], using the individual conceptions as the basis for all analyses.

In 2020, there are 108 universities distributed throughout Germany, with nearly 1.8 million students enrolled [78]. In Bavaria, there are about 246,000 university students [78], 13,000 of whom study at the University of Bayreuth [79]. At the time of data collection, the University of Bayreuth consisted of six faculties, which we all covered in this study.

Especially in Germany, a country with high educational standards, a change in the way plastics and microplastics are handled must be achieved. Accounting for 24% of the total demand, the German demand for plastics is by far the highest in Europe [80]. In 2018, less than 40% of the plastic post-consumer waste was recycled, and up to 60% were used for energy recovery, with a total waste volume of 5 million tonnes [80]. However, with 50%, the recycling rate for the 3 million tonnes of plastic packaging lay above the European average of 42% [80]. Although waste recycling shows a positive trend, Germany’s total volume of plastics remains comparatively high. A rethinking towards more reduction, recycling, reusing, and repairing is necessary to minimize the publics’ impact on the environment.

Our convenience sample consisted of 267 university students. The average age was 20.3 (SD = ± 2.56) years, and 56.6% were female. The sample comprised exclusively of on-campus students of the University of Bayreuth (Bavaria, Germany), representing the university’s six faculties. They belonged to the following scientific disciplines: Natural sciences, humanities, engineering, economics, and cultural sciences. We have included all surveyed students in the analyses as no indications existed to exclude certain respondents.

Participation was voluntary. The students could reject study participation at any time. Due to pseudo-anonymous data collection, it is not possible to assign the questionnaires to individual students. All participating students completed a 15-minute paper-and-pencil questionnaire (approved by the ethics committee of the University of Bayreuth) under the same conditions. At the beginning of the academic term, the students independently answered the questions based on their conceptions and experiences during one of their university courses. All students were given the same questionnaire containing open and closed questions (see Table 2), which allowed a comprehensive assessment their conceptions on topics related to microplastics. More precisely, the questionnaire comprised three open (Q1, Q2, Q3), one closed question (Q4), and a combination of an open and a closed question (Q5a and Q5b). The questions were created explicitly for this study to assess general ideas on microplastics based on topics relevant to fundamental enlightenment. To keep the survey duration as short as possible, we focused on crucial issues for individual’s handling with plastics and microplastics.

**Table 2:** Questionnaire questions.

Question	Wording
Q1	What do you understand by the term microplastics?
Q2	Where do you get your information about microplastics from?
Q3	Name sources of microplastics in the household.
Q4	In which ecosystems are microplastics in Germany? Tick the answer (multiple ticks are possible).
	(a) sea (b) rivers (c) lakes (d) groundwater
Q5a	Assess the potential danger posed by microplastics.
	(a) very dangerous (b) dangerous
	(c) hardly dangerous (d) not dangerous
Q5b	Justify your decision of question 5a.

Q1, Q2, Q3, and Q5b are open questions. Q4 is a multiple-choice question. Q5a is a single-choice question.

The closed questions Q4 and Q5b were used consciously. In Q4, the classification of groundwater as an ecosystem may not be familiar to the non-specialist and may lead to an incomplete mapping of students’ conception of the topic. Regarding question 5a, the risk evaluation, a precise classification along a gradation was beneficial for the validity. For the two closed questions, we determined the number of students who agreed to each answer choice and illustrated them with percentages in the results.

In contrast to the closed questions, the open-ended questions allowed students to describe their personal ideas and thoughts without being guided by predetermined answer choices. Hence, the students’ individual responses to the open-ended questions were the starting point of the evaluation following the qualitative content analysis by Mayring [77]. Within the qualitative content analysis, students’ mentioned terms or explanations formed the basis of a categorical framework, which we constructed independently for each question [81]. This inductive categorization enabled a detailed and accurate recording as well as a homogeneous bundling of the students’ diverse ideas. The category system was progressively refined so that the introduction of gradual subcategories enabled an even more detailed analysis of the responses. The developed coding guidelines included a clear category definition and an anchor example from the student responses for each question to ensure transparent categorization. Table 3 shows an excerpt of the coding guideline of Q1. Since the students’ conceptions about a topic were quite multidimensional and partly conceptually multilayered, a student’s answer could be assigned to several categories simultaneously. After determining and setting all statements to the appropriate categories, the category assignments were quantified. This quantification allowed an accurate determination of the number of students holding a particular conception about every single topic concerning microplastics. Hence, also the students’ conceptions on the open questions are given in percent in the results.

**Table 3:** Exemplary coding guideline of Q1.

Category	Definition	Anchor Example
Small plastic particles	Microplastic is described as small plastic. These include terms such as *small/microscopic plastic particles*, *plastic particles not visible to the eye*, and general references to the small size of plastics.	ID 230: “**Small plastic particles.**”
Plastic	Microplastic is described as *plastic* or *small amounts of plastic*. No reference is made to the size of the plastic.	ID 211: “**Plastic** in waters that pollute waters and are taken up by animals.”
Primary MP	In the description of the term, reference is implicitly made to primary microplastics, *e*.*g*., by mentioning the direct production of small plastics or their presence in personal care products.	ID 207: “Smallest plastic particles such as **peeling grains.**”
Secondary MP	The term’s description implicitly refers to secondary microplastics, for example, by addressing the defragmentation of plastic into microplastics.	ID 232: “Microscopic plastic particles that are **created when plastic disintegrates**. This is extremely harmful to organisms.”
Effects	In the description of the term, the effects of microplastics on the environment and humans are mentioned. Among the listed topics are environmental damage, damage to health, presence in the environment, in living beings and in food, indigestibility, and the difficulty of removing microplastics from the environment.	ID 4: “Tiny little particles of plastic. **Harmful to the environment.**”

Relevant statement parts for categorization in bold.

The first author categorized all data of the open questions. To validate the categories, a randomly selected 20% subsample of the data was reanalyzed after one year by the first author to estimate intra-rater reliability and by another independent, nonpartisan person to receive inter-rater reliability statistics. The intra- and inter-rater Cohen’s kappa coefficients were calculated for all four open questions (see Table 4) [82]. Cohen’s kappa coefficients indicate the measure of agreement between different people, also called raters, on identical rating systems [83]. The higher the calculated score, the higher the agreement on the category system for the individual answers. Cohen’s kappa value was calculated by examining the percentual accordance of raters’ categorization of data input [84]. Thereby the measurement considers the statistical probability of random agreements, reducing the weight of the value systematically [84]. Cohen’s kappa scores ranged between.86 and.97, indicating an ‘almost perfect’agreement between the raters, which is, following Landis & Koch [85], reached above the value of.81.

**Table 4:** Cohen’s kappa scores for intra- and inter-rater reliability of questions Q1, Q2, Q3, and Q5b.

	Cohen’s Kappa Score	
Questions	Intra-Rater Reliability	Inter-Rater Reliability
Q1	0.96	0.86
Q2	0.94	0.96
Q3	0.93	0.95
Q5b	0.97	0.94

## Results

First, we show the results of the two closed questions on microplastic the sinks in German waters and the risk evaluation. Afterward, we explain the results of the open questions, which we quantified by using the categories we created from the students’ answers. For the open questions, we summarized all answers belonging to the category ‘expression of ignorance’and ‘inadequate answer’as ‘no answer’. Single conceptions held by less than 3% of the students were conflated in ‘other’. We omitted the categories ‘no answer’and ‘other’in the Figures for a better overview. The frequencies in the Figures refer to the number of students whose answers can be assigned to the respective category.

### Students’ conceptions of microplastic sinks in German waters

**Fig 1:**
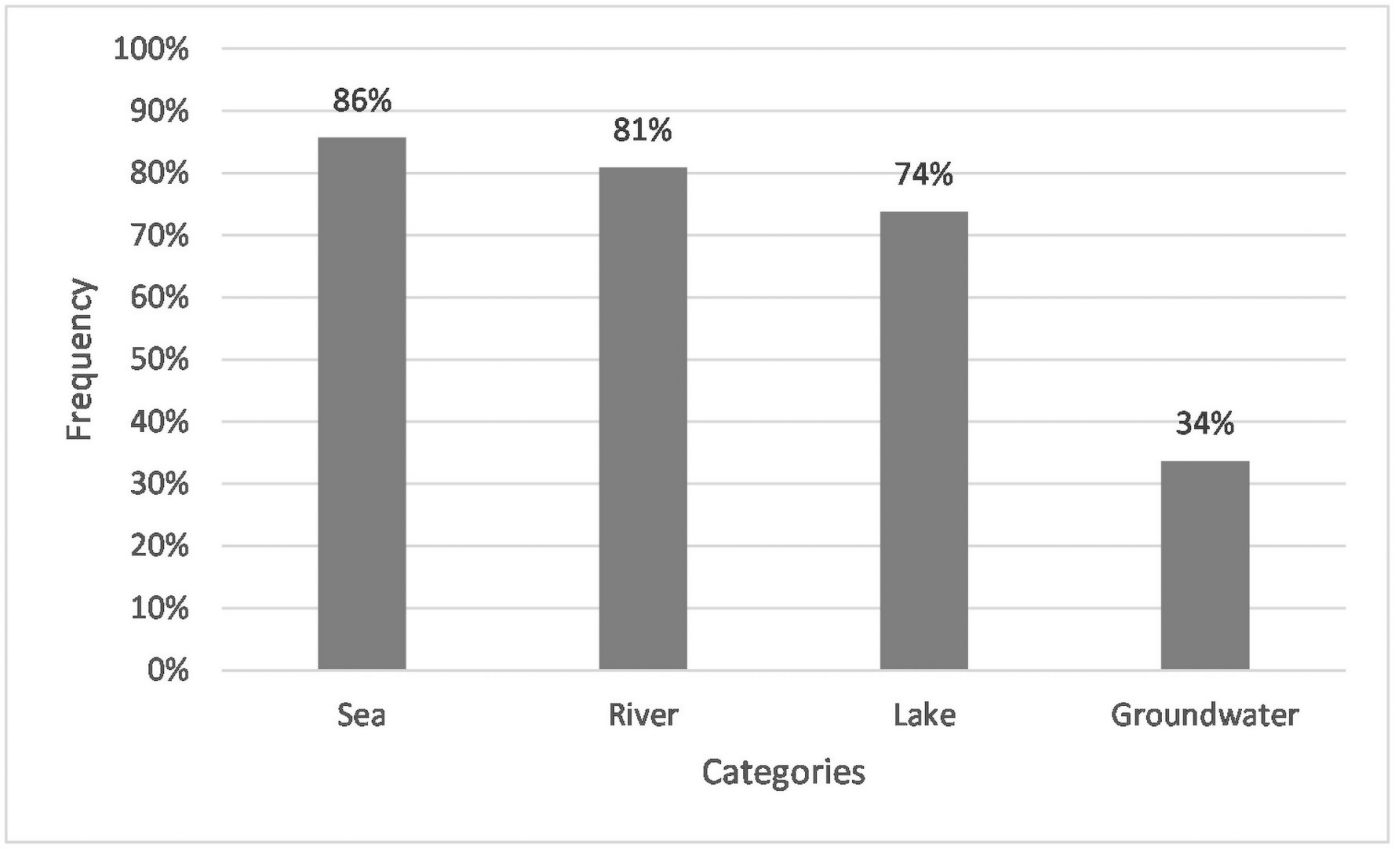
Frequencies of students whose answers assigned to the corresponding categories for the closed Q4: “In which water ecosystems is microplastics in Germany? (a) sea, (b) rivers, (c) lakes, (d) groundwater.” N = 267. Closed question with predetermined answers.

In this multiple-choice question, the closed response format offered students four aquatic ecosystems for selection (see Table 2). 86% of the students thought that the German sea contained microplastics. 81% of those surveyed considered German rivers to be polluted. 74% of the students regarded lakes as burdened, and for groundwater, 34% of the students indicated a microplastic load (see Fig 1).

### Students’ risk evaluation and justification

**Fig 2:**
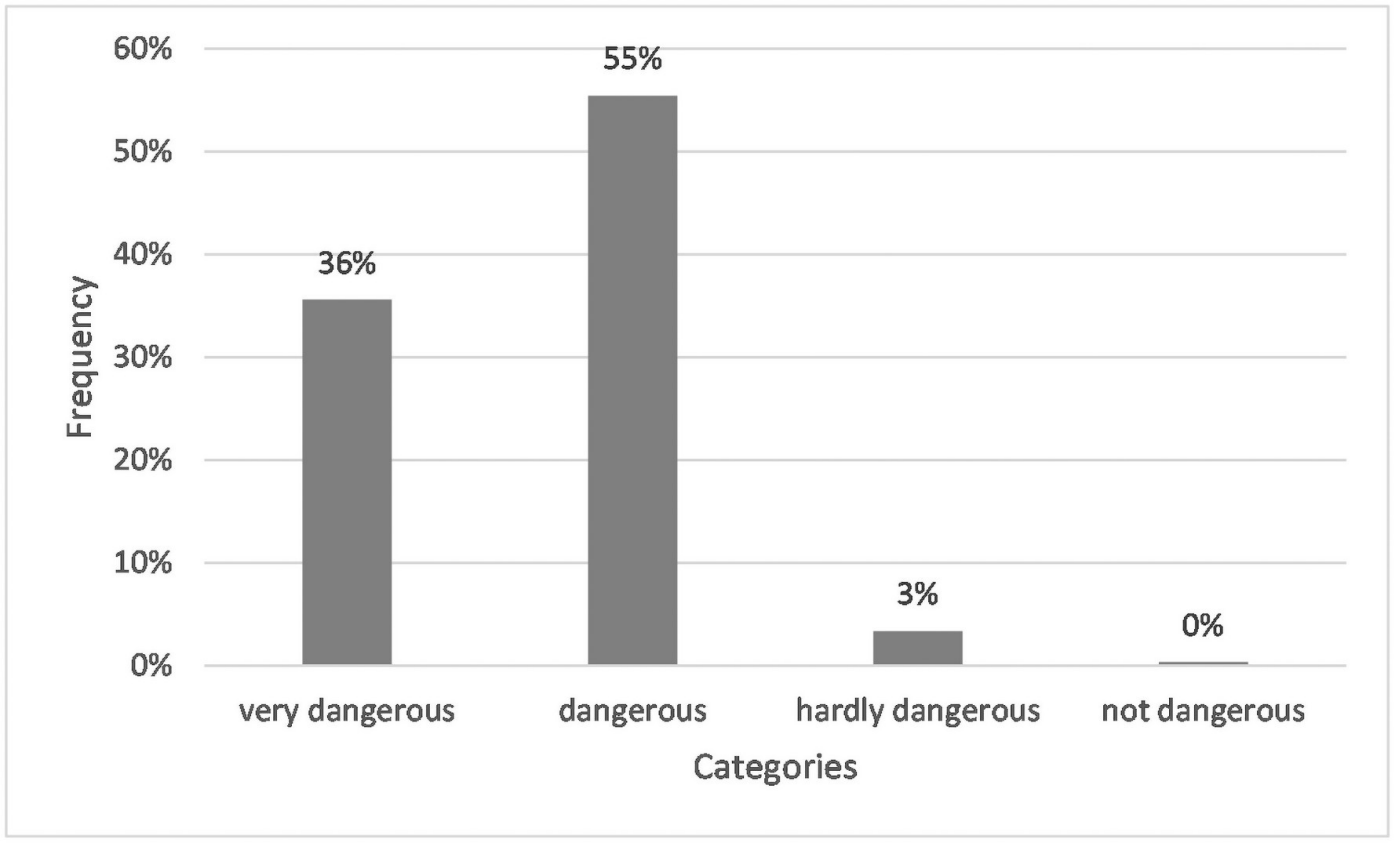
Frequencies of students whose answers assigned to the corresponding categories for Q5a: “Assess the potential danger posed by microplastics. (a) very dangerous, (b) dangerous, (c) hardly dangerous, (d) not dangerous.” N = 267. Closed question with predetermined answers.

The closed question on risk evaluation (for response options, see Table 2) showed that 36% of the students classified microplastics as very dangerous, and 55% considered microplastics as dangerous. Solely 3% sorted microplastics as hardly dangerous, and nobody ranked it as not dangerous (see Fig 2).

**Fig 3:**
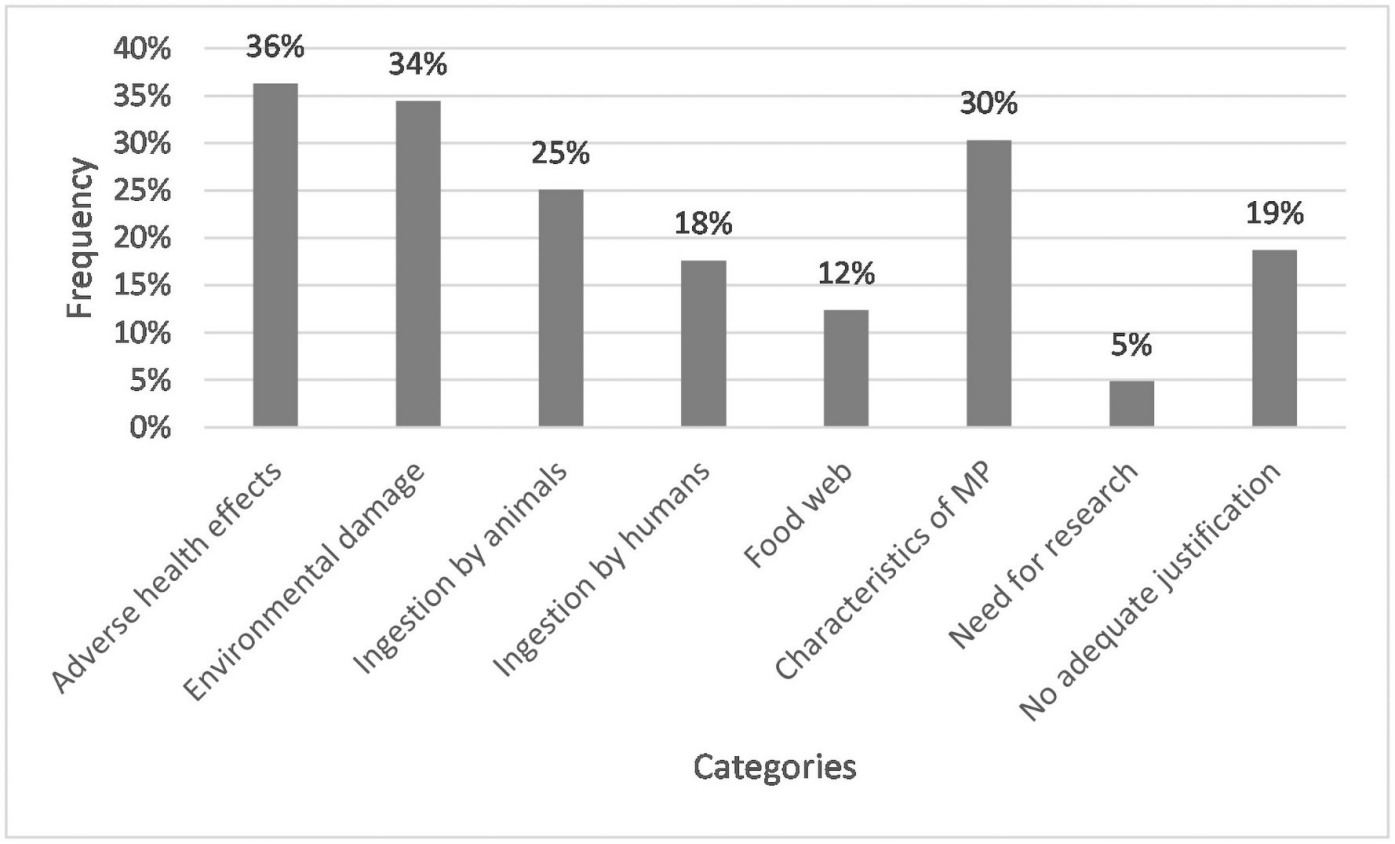
Frequencies of students’ answers assigned to the corresponding categories for Q5b: “Justify your decision [of Q5a].” N = 267. Open question with categories formed from students’ answers.

The following open-ended question was designed to capture the student’s rationale for their risk evaluation. The justification of the stated risk evaluation provided diverse explanations. 36% of the students indicated general health hazards for all living organisms (*e*.*g*., resulting in the accumulation in tissues). 34% named environmental damage like ecosystem contamination (*e*.*g*., water pollution) or negative influences on plants. 25% stated ingestion by animals (especially by marine life like fish), 18% described uptake as food by humans, and 12% listed stress of the whole food web to explain their risk evaluation of microplastics. Moreover, 30% of the students named microplastics’ properties (*e*.*g*., characteristics stemming from additives or adsorbed substances, small size, indigestibility, non-degradable) to justify their risk evaluation, like student ID 98 did: “Plastic is not biodegradable. Therefore a lot of plastic will accumulate over a long period of time. Especially if the plastic parts are very small, hardly visible, they will probably be ingested quickly without intention, which is probably more harmful than healthy.” Finally, 5% claimed a need for further research as an explanation for their risk evaluation. Regarding the need for research, it was noted that more research is required in this area to weigh up the consequences and risks. Students’ responses often contained serval categories simultaneously, as was the case of student ID 155: “Plankton stores plastic, which is passed on in the food web and in the end it also affects humans. Furthermore, medical and biological consequences are not foreseeable, and there is no way to remove the garbage.” This student’s justification comprised the categories ‘ingestion by animals’, ‘ingestion by humans’, ‘food web’, and ‘need for research’. Besides these detailed answers, 19% left their risk estimation unfounded (see Fig 3).

### Students’ conceptions of the term microplastics

**Fig 4:**
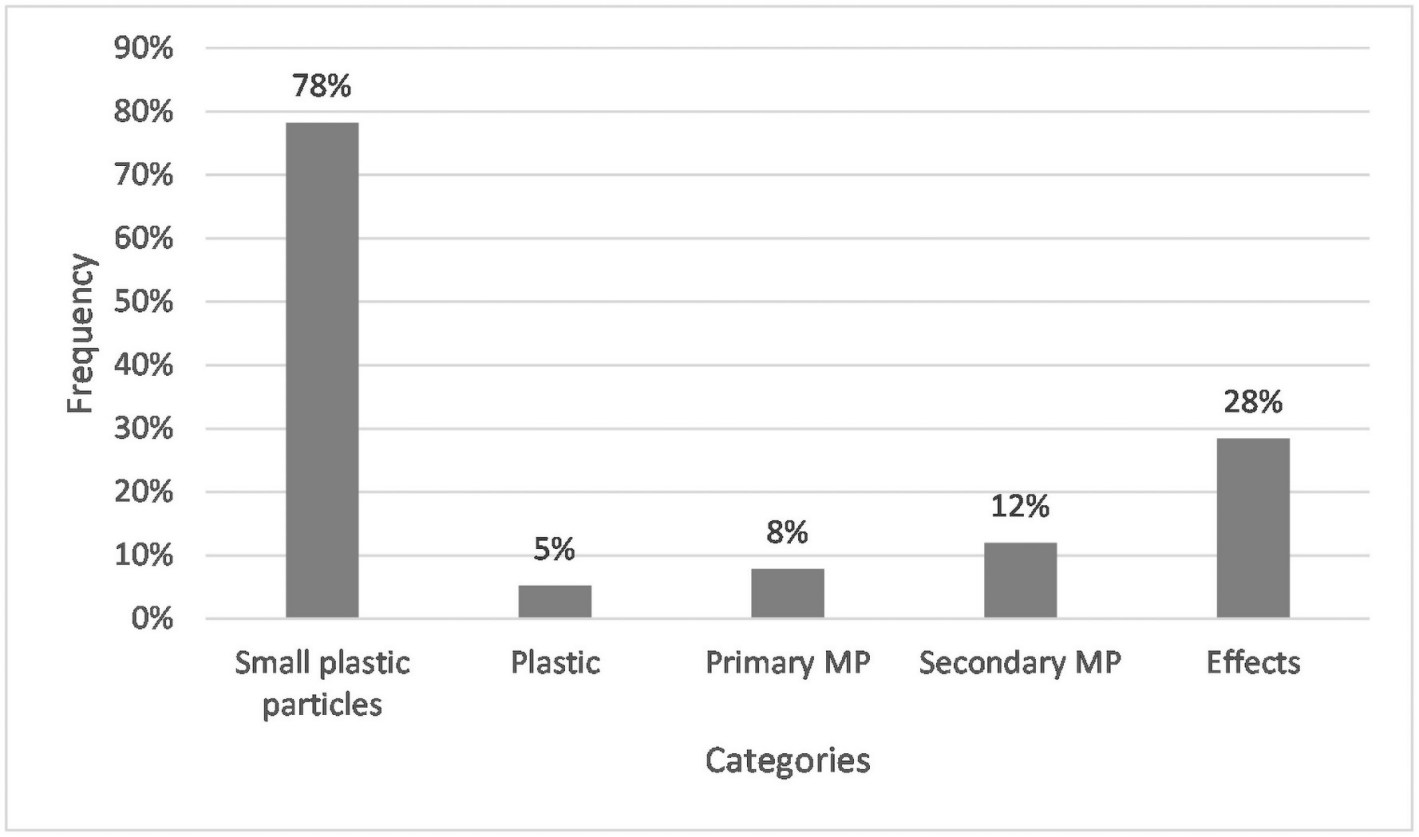
Frequencies of students whose answers assigned to the corresponding categories for Q1: “What do you understand by the term microplastics?” N = 267. Open question with categories formed from students’ answers.

78% of respondents classified the term microplastics with reference to small plastic particles (see Fig 4). 5% of the students ranked microplastics as plastic. In addition to a basic classification, some students included more profound information in their definitions. 8% respectively, 12% explained primary and secondary microplastics, as the answer of student ID 194 shows: “Very small plastic particles. These can already be present as such, *e*.*g*., in cosmetics, and thus get into the environment or the human organism during or after use, or plastic waste, especially in the oceans, is broken down by mechanical action and thus becomes microplastic.” This answer also demonstrates that a student’s response to one question can contain several categories simultaneously. In this case, the categories ‘small plastic particles’, ‘primary microplastics’, and ‘secondary microplastics’.

Furthermore, almost every third student (28%) discussed effects, although these were not asked. In some cases, the students only briefly addressed the consequences, as student ID 4 did: “Tiny little particles of plastic. Environmentally harmful”, who pointed out the environmental damage. Others discussed the adverse effects of microplastics in more detail, like student ID 145: “Small plastic parts which […] can be found everywhere (food, water, sand). It is speculated that this, i.e., the ingestion of microplastics, can cause health risks.” Hence, the addressed negative consequences included topics like pollution, ingestion by organisms, accumulation in organisms, indigestibility, durability and lack of degradation.

### Students’ sources of information

**Fig 5:**
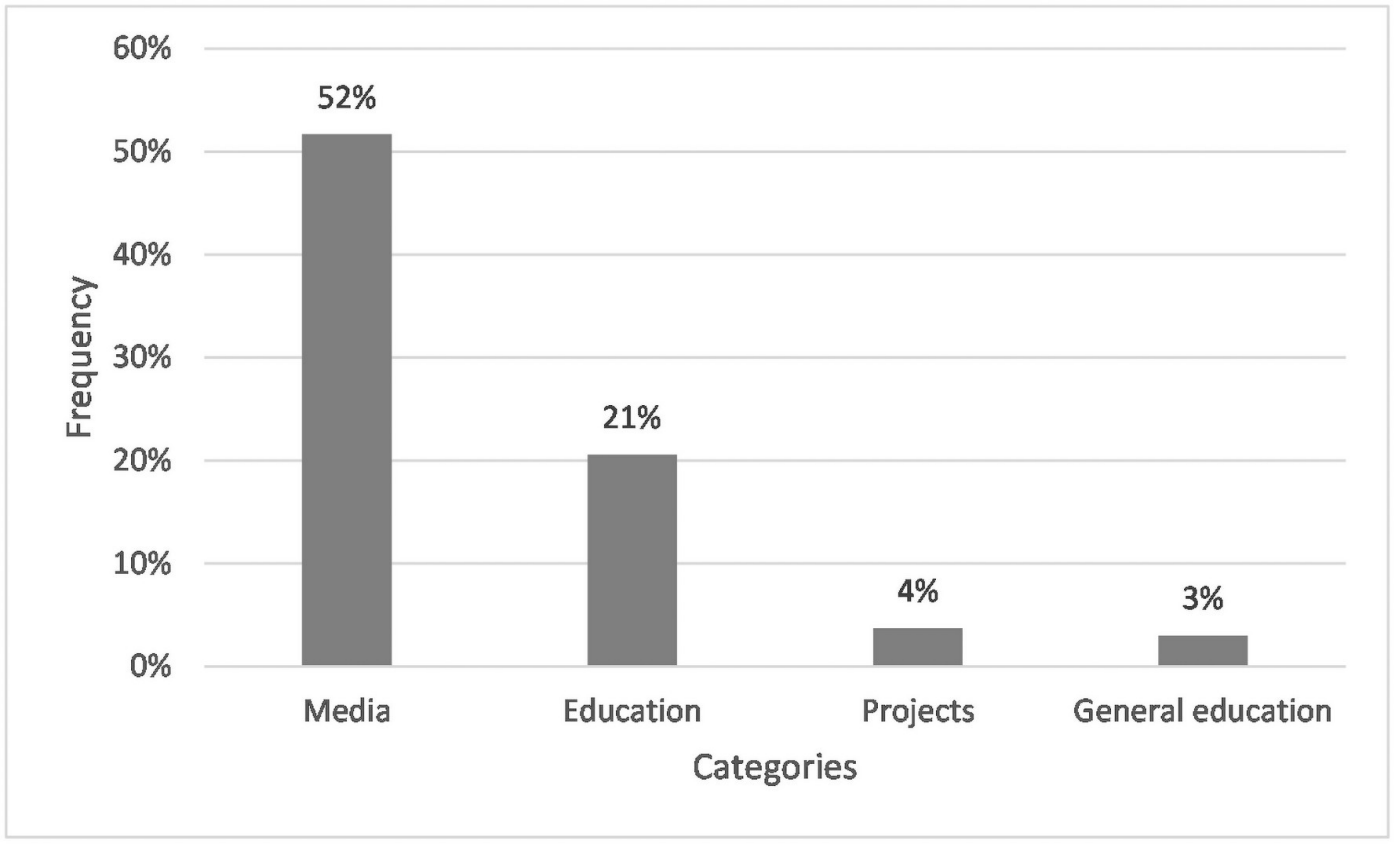
Frequencies of students whose answers assigned to the corresponding categories for Q2: “Where do you get your information about microplastics from?” N = 267. Open question with categories formed from students’ answers.

52% of the respondents indicated the media as their source of information. Another 21% named educational institutions, and 4% projects and nature conservation organizations as their source of information. 3% of the respondents considered it part of their general education (see Fig 5).

**Fig 6:**
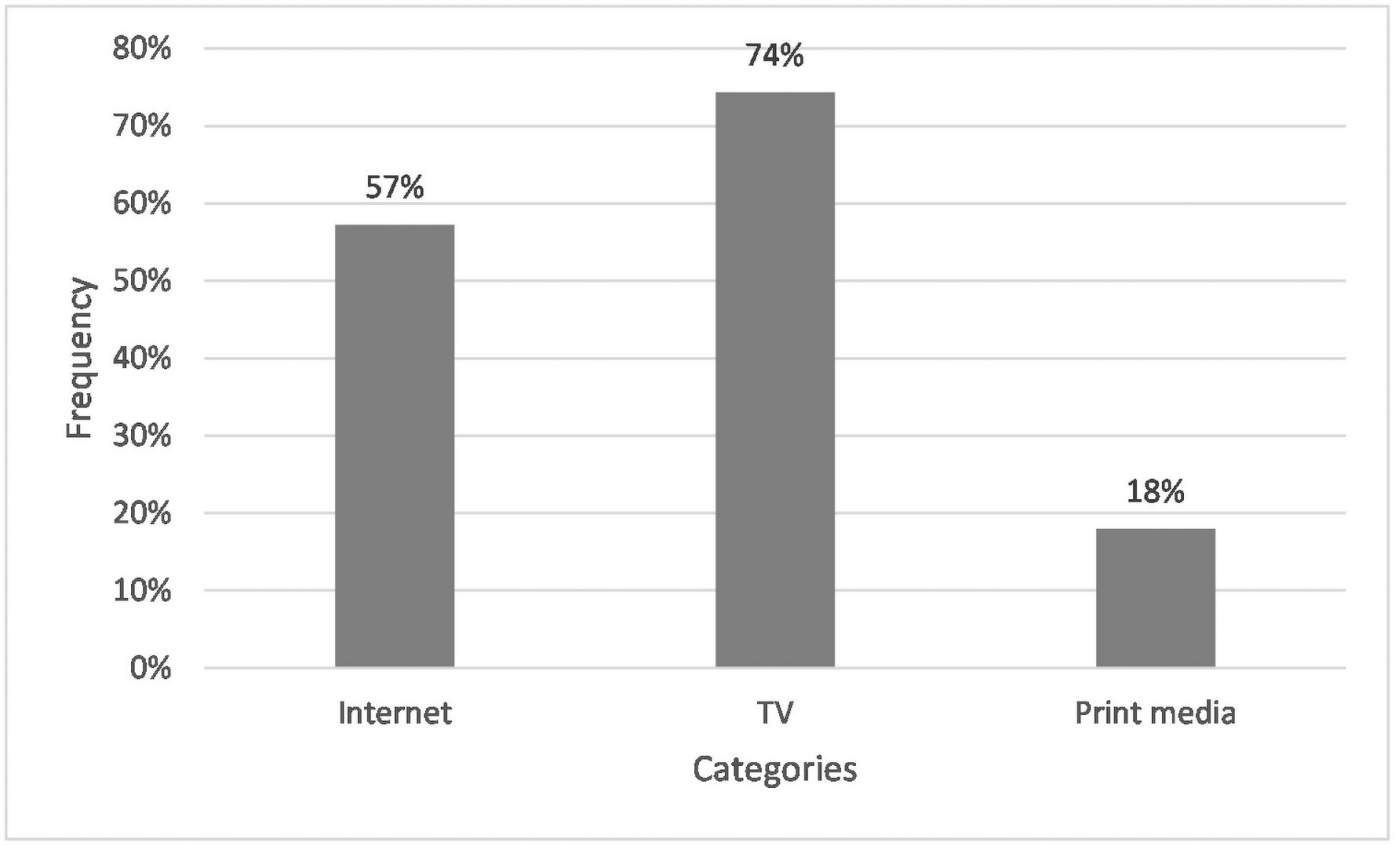
Frequencies of students whose answers specified the term ‘media’in Q2: “Where do you get your knowledge about microplastics from?” n = 117. Open question with categories formed from students’ answers.

85% of those who listed media further specified this category by naming television, Internet, and print media (see Fig 6). Television (76%) proved to be the essential source of information in the media field. Within the television subcategory, respondents stated documentaries and news as sources of information. On the Internet (57%), social media and individual information retrieval via google were relevant. Newspapers were the basis for information for the print media (18%).

**Fig 7:**
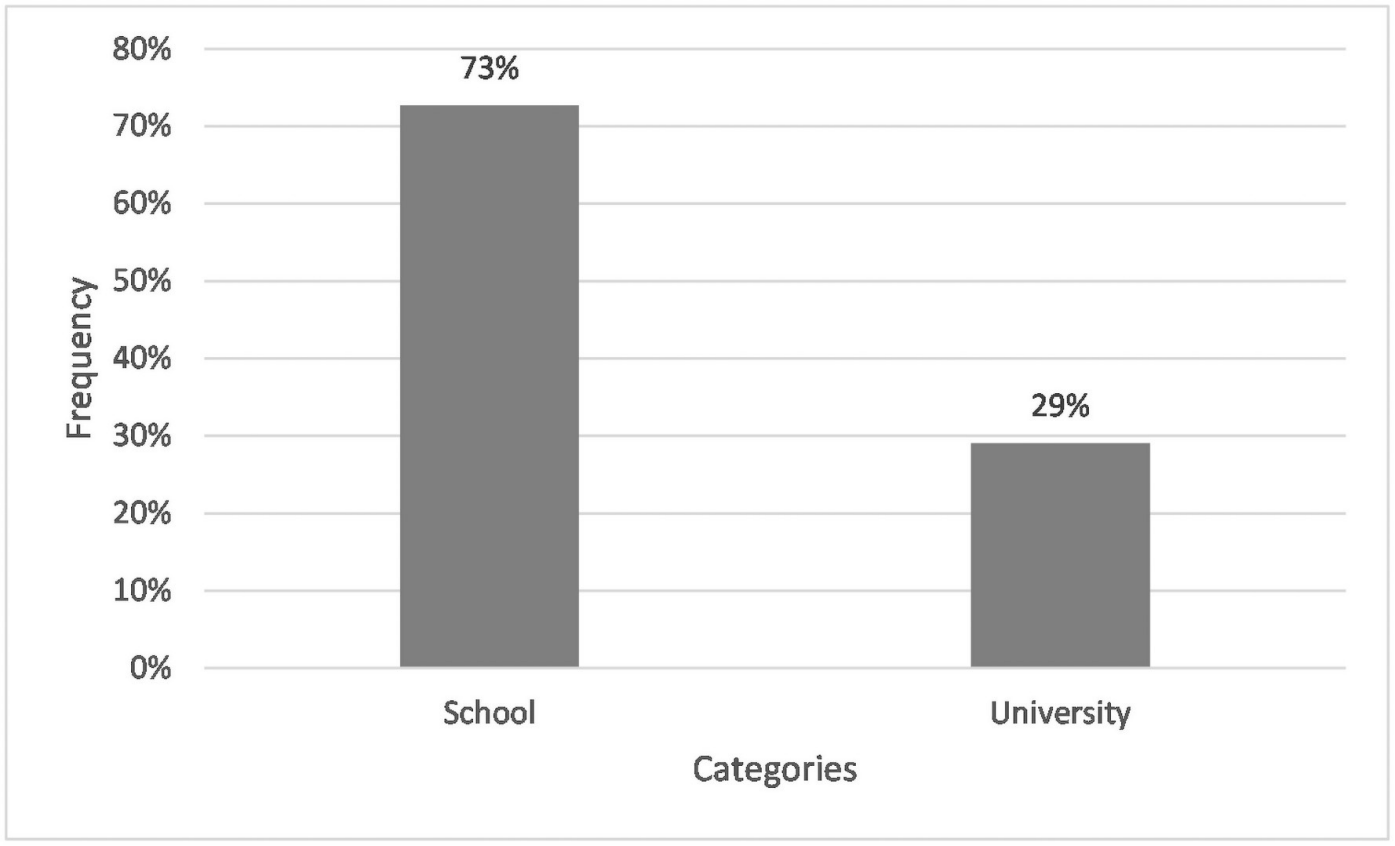
Frequencies of students whose answers specified the term ‘education’in Q2: “Where do you get your knowledge about microplastics from?” n = 55. Open question with categories formed from students’ answers.

When taking a closer look at the subcategories of education, schools (73%) were the most important source of information, ahead of universities (29%). It should be noted here, however, that merely 21% of the students in total named educational institutions as a source (see Fig 7).

### Students’ conception of microplastic sources in the household

**Fig 8:**
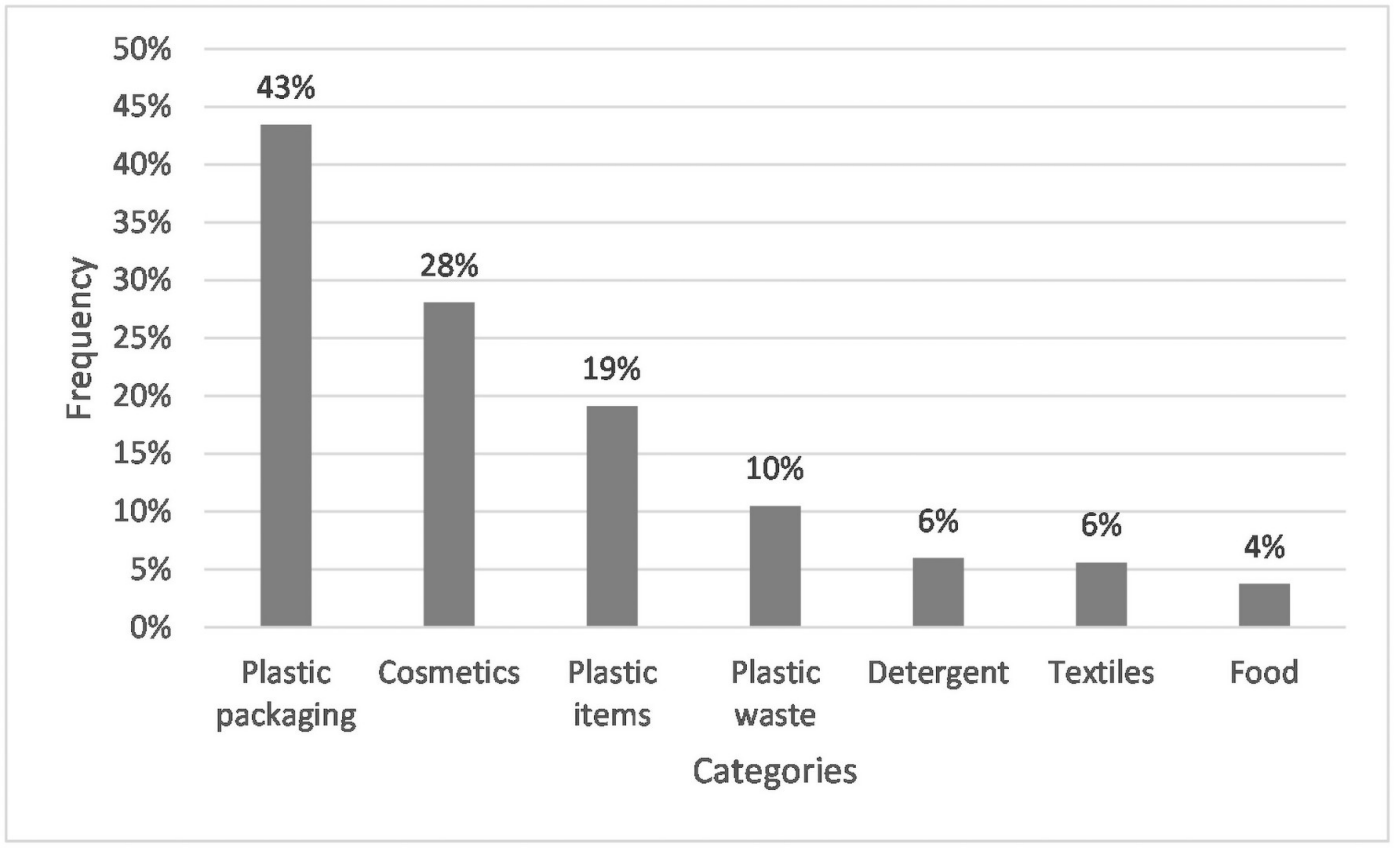
Frequencies of students whose answers assigned to the corresponding categories for Q3: “Name sources of microplastics in the household?” N = 267. Open question with categories formed from students’ answers.

As a possible source of microplastics in the household, plastic packaging (e.g., plastic bags, plastic bottles) was mentioned by 43% of the students. Almost one in three (28%) named various cosmetic products (*e*.*g*., make-up, shower gel), 19% mentioned diverse plastic objects (*e*.*g*., kitchen utensils, toys). A smaller number of students (10%) listed plastic waste (containing references to inadequate waste disposal), 6% of the respondents named detergents and textiles, and 4% food in their answers (see Fig 8).

## Discussion

The students’ input on microplastics revealed expected as well as surprising denominations. In general, the students were familiar with the broad outlines of microplastics, and the majority understood the term microplastic to mean small plastic particles. Surprisingly, conceptions strongly associated microplastics with adverse effects, consequently classifying them as dangerous. This evaluation may stem from their primary source of information, which is not educational institutions but the media, where simplification of scientific findings or interpretative frames might influence the perception of danger [86]. The risk estimation revealed a diverse picture of justifications, including scientifically sound statements and explanations that have not yet been thoroughly scientifically explored. Hence, the conceptions uncovered some superficial understandings of the topic. The study also revealed results, which were quite in line with our expectations: Many students mentioned plastic packaging, and even cosmetics, despite its small proportion of the overall problem, as household sources [87]. Other sources, *e*.*g*., fibers from hygiene products and plastics in organic waste, were ignored. Concerning sinks in German waters, compared to the ocean, lakes, and rivers, relatively few students suspected microplastic contamination of groundwater. In the following, we discuss the major outcomes of conceptions, beginning from proposed blind spots to the role of media and recommendations for action in science communication. Since the present study was conducted among students, the results can only draw a conclusion about this special study group. The results do not permit any generalization to students as such or to society in general. Thus, the following discussion on students’ conceptions about microplastics should be read accordingly.

### Blind spots in the household and unawareness about groundwater pollution

Some conceptions indicate a need for action concerning education. Taking Table 1 into consideration, there are starting points for imparting microplastic sources in the household, which are not yet in the students’ consciousness. Only a few students mentioned plastic items, plastic waste, detergents, textiles, or food products. Similar results were obtained in the study by Deng et al. [88], in which the respondents were familiar with conventional plastic products such as plastic bottles but less familiar with paints or textile fibers. Also, in this study, none of the students was aware of the release of fibers from hygiene products (*e*.*g*., feminine hygiene products or cotton swabs) or plastic items in organic waste. Insufficient cleaning processes in sewage treatment plants [10] allow hygiene products’ components to enter waterways after improper disposal in the toilet. Hence, correctly disposed hygiene products pose less of a problem for the environment. Similarly, household plastics in organic waste, predominantly packaging, may enter the environment on agricultural sites and gardens as organic fertilizer from biowaste fermentation and composting [89]. In both cases, education on proper disposal could achieve a reduction of microplastic inputs.

Moreover, answers to the closed question on microplastic polluted water ecosystems revealed another blind spot of the students. While they seem to be familiar with microplastics in the sea (ticked off by 86%), lakes (74%), and rivers (81%), comparatively few students stated microplastic pollution of groundwater (34%). This result is in accordance with Re [90], who argued that microplastics in groundwater sparsely get scientific and political consideration. Rivers [91], lakes [19], and especially the sea [14] gained a lot of scientific and thus media attention.

Different studies have already found results in water’s thematic context, giving impulses for approaching the topics through education. Fremerey et al. [92] surveyed 10^th^ graders and undergraduates’ conceptions about drinking water. They found some unexpected, persistent alternative conceptions: Both pupils and students believed drinking water to be purified in sewage treatment plants. Obviously, fundamental understandings of sewage treatment plants and waterworks processes were confused, leading to persistent alternative conceptions on the purification process of drinking water. Furthermore, studies can reveal blind spots like it was the case for the term ‘virtual water’[92]. In a study on Ecuadorian students, Liefländer et al. [93] detected unfamiliar terms concluding that education about these terms is necessary to avoid overgeneralization. Hence, those gaps are subject areas that need to be explained in very basic terms to prevent students from explaining these concepts based on their own experiences, leading to superficial approaches and interpretations that differ from scientific perspectives. Schmid’s & Bogner’s [94] study on students’ conceptions on water reuse supports this claim. Although knowledge about new technologies of water reuse was missing, students expressed concern about water quality. Schmid & Bogner [94] concluded well-thought outreach activities as necessary to create acceptance. Alternative or missing conceptions on special topics are essential clues for educators and experts in the field. Education on these contents must be handled in a particularly sensitive and skillful way to enlighten students about unknown topics and approximate their conceptions to scientific ones.

### Reasonable conceptions about what microplastics are

The students classified microplastics as small plastics. Hence, the thematic area concerning microplastics is quite common among them. Our result contrasts with Deng et al. [88], finding this term mostly unknown among the participants. Microplastics’ division into primary and secondary microplastics succeeded only implicitly, *i*.*e*., without mentioning the terms primary and secondary. Although the subcategories were not explicitly named, it is still clear that they are aware of different origins of microplastics, namely those that were intentionally produced for usage (primary) and those that arise through degradation (secondary). This comprehension also becomes evident in the question of microplastic sources in the household. Even though only a small number of the students implicitly mentioned primary and secondary microplastics in Q1, the answers in Q3 show that a larger part is aware that these two types of microplastics exist. In their responses, the students mentioned cosmetics (belonging to primary microplastics; [5]) and plastic packaging or plastic objects, which are merely a microplastic source when used or otherwise disintegrate into tiny pieces smaller than 5 mm (secondary microplastics; [6]). All answers that fall into the categories’ plastic packaging,’ ‘plastic objects,’ ‘plastic waste’ and ‘textiles’ indicate students’ awareness for microplastics’ creation from plastic, hence secondary microplastics. The results suggest that the concepts of primary and secondary microplastics are known among the students; however, they lack the corresponding technical term. Here, a term clarification would be appropriate to enable students to title the concepts they already have.

### Plastic packaging as the main source in households

With 43%, plastic packaging was the most frequently mentioned source in the household. Plastic packaging can, of course, be attributed to plastic waste (10%). However, at this point, it was explicitly categorized individually because of the high number of responses that explicitly addressed plastic packaging. A level of detail that we did not want to lose. Estimations on the origin of plastic (debris) in oceans show roughly 80% as land-based, while approximately 20% deriving from maritime activities like fishing [95, 96]. Especially in Germany, the demand for plastics is not reducing but remains high compared to the other European countries [80]. Students’ focus on plastic packaging is hardly surprising, given that it represents the majority (namely 39.6%) of European plastic demand [80]. Hence, packaging material presents a substantial part of plastic litter and, accordingly, plastics in the environment. In light of these numbers, the abundant mentions of plastic packaging are justified and a crucial starting point for reducing plastics and microplastics’ entry into the environment.

The apparent understanding of plastic packaging as a microplastic source in private households among the students can be an anchor point in the process of behavioral change. Leire & Thidell [97] described in their study missing mindfulness as responsible for consumers’ lacking connection between purchasing decisions and environmental consequences due to a lack of awareness. A study by Hartley et al. [98] reported that participation in a classroom module on marine litter led to improved students’ self-reported environmentally friendly behaviors, which were also passed on to friends and family. Hence, education on environmentally relevant topics possesses a great potential to increase pro-environmental behavior concerning plastics and microplastics. In line with these findings, the central goal in the future is to make learners aware of how their chewing choices and plastic consumption directly impact plastic and microplastic pollution in nature [99].

Since the plastic waste problem is a problem of human behavior and not solely of plastics’ characteristics [100], these results underline educational initiatives’ fruitfulness and the necessity for additional programs on everyone’s responsibility.

### Overrepresentation of cosmetics

Also unsurprising, personal care products (especially cosmetics) were the second most cited microplastic source in households. In a study of 2016, the topic was still unknown among students [101]. The relatively strong focus on personal care products among the students we surveyed can be explained by the increased attention in scientific studies [87], the large media presence [86], or the targeted advertising measures of the cosmetics industry. Although care products are currently attributed a relatively low relevance to the overall problem of microplastics [87], an awareness of this source in one’s household is nevertheless valuable.

### Perception of microplastics as dangerous hazard

Students perceive microplastics as very dangerous or dangerous. The proportion of those who considered microplastics to be barely dangerous was vanishingly small. No one considered them to be harmless. Reasons were diverse, ranging from effects on humans, animals, and ecosystems to microplastics’ characteristics and the demand for more research. In contrast to the surveyed students, the respondents of Deng et al. [88] showed a more pronounced anthropocentric view of plastic pollution. From a selection of negative effects of plastics, the respondents felt most affected by the city’s pollution, i.e., their personal, man-made environment. Some justifications were scientifically sound and well-studied; others included topics on which scientists themselves still disagree or are not yet researched. While scientists agree on the ingestion of microplastics by many organisms [25], the consumption’s health consequences are still being investigated and not yet thoroughly understood. Some studies point to negative consequences like inflammation [102], while other studies cannot show measurable effects [103]. Further eco-toxicological studies need to broaden the assessment of the extent of microplastics’ impact on health.

The probably most critical point to note was the missing response rate of 19% of the students when they were asked to justify their risk evaluation. Given that only 3% considered microplastics as hardly dangerous and no one saw any danger coming from it, the question arises whether the students are eventually oversensitized by the topic without thoroughly understandying why. Almost one-third of the students depicted microplastics’ effects in their definition, although these were not required in the question. This rate indicates a strong thematic link between the topic of microplastics and its effects. The fact that the characterized consequences were purely negative signals a strong negative association of the respondents with microplastics, especially since none of the respondents named even one positive property or influence. Such a negative connotation of microplastics in connection with the lack of benefits was also reported by Kramm et al. [104]. The observed high sensitivity to microplastics’ hazards possibly arises from the representation of risk in the primary information source, the media [105]. In this context, the framing in media reports may play an important role [86]. Framing emphasizes individual, selected aspects within a communication process, which the sender chooses to color the facts in a manner intended by him [106]. Scientific uncertainties are often omitted in the media due to simplifying scientific findings or biased reporting, suggesting a higher probability of risks stemming from microplastics than objectively surveyed [86]. Different risk conceptions may lead to an overestimation of risks by the public. While scientists classify risk as “the probability of a negative outcome”, the public understands risk as “the uncertainty of a negative outcome itself” ([86], p. 1). Thereby a different evaluation of scientific findings on potential risks dominates the public mind. Due to the strongly negative attitude towards microplastics, education should also address plastics’ positive properties as problem solvers in modern society, *e*.*g*., for food safety or application in the medical field [107], to avoid a one-sided view of plastics.

### Media as the primary source of information: School is falling behind

As media can inform many people uncomplicatedly and directly, non-surprisingly media perceived a great relevance (named by 52%), which is in accordance with Deng et al. [88]. In contrast, the education sector as a source of information was scored by just 21% of respondents, while the university even played a minor role than schools. Although the media landscape has changed a lot and media use has undoubtedly grown since then, already in 1987, Blum [108] reported schools as less critical than mass media (TV, radio, private reading) as sources of students’ knowledge and beliefs. Nelkin ([109], p. 2) concluded that science is understood “less through direct experience or past education than through the filter of journalistic language and imagery”. Several other studies on student conceptions already found media as an essential source [110, 111]. Our results confirmed these results, which is of concern, as the topic should be given an important place in schools and at university (especially in science courses) due to its topicality and global impact. The low number of university mentions may lie in the young semester and in the large number of study programs surveyed, covering not solely science classes. Hence, the numbers of pure science majors might look different.

Looking at the students’ information in more detail, the high proportion of TV in media can be seen as positive, as they cited news, reports, etc., as a source. According to Brossard [112], the Internet has a growing relevance in procuring information, which is also reflected in our results. The Internet can be a reliable source if it is used for research on high-quality sites. The growth in the usage of social media has undoubtedly proceeded in the last two years. This trend must be regarded with caution since content, as long as it does not come from official organizations or scientists, is hardly checked and quickly distributed.

Media have the potential to form scientific knowledge and thereby shape public conceptions [113]. This is also the case for topics on plastics and microplastics. Therefore, the way information about plastic pollution is presented through the media can influence society’s understanding [114]. Next to positive aspects such as speed, timeliness, and range, the media reporting also holds disadvantages, *e*.*g*., when the coverage quality suffers from exaggeration, oversimplification, or misrepresentation [115]. Hence, media can also contribute to disseminating and perpetuating alternative conceptions [116], which possibly become entrenched in the public’s mind by repeated reporting. An example of a persistent alternative conception in connection with plastic pollution is the Great Pacific Garbage Patch, which is anchored in the public’s mind as a closed garbage patch [114, 117] and most likely derived from multiple media reports on the subject, which often used images of plastic-flooded rivers to illustrate the point. Contrary to this popular notion of a carpet or island of plastics, the Great Pacific Garbage Patch is instead a collection of individual plastic items in the North Pacific Ocean [117]. Science communication to the public with appropriate tools is needed to clarify that the Great Pacific Garbage Patch is rather a matter of individual plastic fragments accumulating than a plastic island. Next to the Great Pacific Garbage Patch, various issues related to microplastics exist for which alternative conceptions have been formed in public. Hahladakis [117] attempts to educate and clarify these alternative conceptions concluding education on the topic essential.

Also teachers are not immune to alternative conceptions [116]. Before lessons, teachers should reflect on their own perceptions and check them with the help of several high-quality sources to identify deviations [118]. Holding alternative (non-scientific) conceptions, teachers possibly may not recognize them in their classroom [111] or even impart their own to their students. Thus, students and teachers might benefit from reliable information from first-hand sources other than the media. Against this background, schools and universities should much more become places that provide up-to-date information on topics relevant to the day and opportunities for students to exchange ideas with teachers, scientists, and fellow students, questioning their own perceptions and discussing divergent views. Strengthening the educational sector would facilitate young adults’ responsibility as a well-informed part of society.

### Science communication desired

The demand for professional science communication on different media channels and educational institutions is apparent: Transferring the knowledge directly from the scientific community to the general society is vital but needs a language that both sides understand and decode similarly. Burns et al. ([119], p. 191) consider the “use of appropriate skills, media, activities, and dialogue” as a prerequisite for successful science communication. Science education is conceived as the foundation for science communication: The greater people’s familiarity with science, the better the understanding of the communicated content will be [120]. To render scientific ideas intelligible, a layperson’s perspective may help recognize the gap between both knowledge levels [120]. This is where our study results come in. Knowledge of students’ conceptions on microplastics can be valuable starting points for tailor-made science communication initiatives addressing pertinent ideas and excluding already well-established and understood topics. Subsequently, contextual and conceptual adjustments on the subject matter, which scientists aim to convey, have to be performed. Thereby, among other things, the adaptation of vocabulary and jargon is fundamental to creating effective communication with the public [115]. Obviously, the term microplastics has arrived in everyday lives and natural language use. However, special attention to vocabulary and framing in microplastics’ consequences and risks is delicate. Furthermore, media and actions have to be tailored to the target audience [119]. Scientists should much more enter the relevant media channels like TV and the Internet to reach a broad audience. This also includes close cooperation with journalists by sufficiently informing them about the main statements, misunderstandings, and misinterpretations. As successful science communication is based on a two-way dialogue that facilitates communicative interaction [119], disseminating knowledge and exchanging opinions via social media without intermediary journalists may be a suitable tool. Indeed, successful science communication is beneficial for researchers and the general public [121], helping people make informed decisions concerning public and private lives [109, 122]. If scientists succeed in this challenging and vital task, a science-literate person will possess profound knowledge to participate in scientific discussions and act wisely.

### Need for action

Research on microplastics enlarges continuously, which accordingly changes the knowledge levels of scientists [9]. There is agreement among scientists that plastic debris is everywhere in the world [123]. Furthermore, scientific findings revealed that the ubiquitous occurrence definitely has impacts, *e*.*g*., by entangling organisms [25], by changing soil properties [56], by the colonization on its surface [124], or by the ingestion of it [50]. Although potential consequences need further research [125], the question remains how much knowledge is necessary to initiate changes. Knowing that a human-made substance is and will be strictly speaking everywhere should be enough to rethink society, public policies, and industry towards a circular economy and a reduction in consumption. The constant expansion of knowledge about microplastics also indicates the need for action in the educational sector.

The speed of new scientific findings shows that the public’s current state of knowledge is never satisfactory but should steadily be expanded [126]. Efficient science communication provides the general public with information “that they need in a form they can use” ([120], p. 14038) while guaranteeing that society is not left behind at an outdated state of knowledge but continuously kept informed about new developments. Dissemination through modern media channels by experts in the field can transport scientifically correct information to a broad audience [127]. Next, scientists’ outreach activities would be appropriate methods to impart scientific knowledge directly to students [121]. If the responsibility is handed over to educators in different educational institutions, it must be ensured that they also have up-to-date scientifically sound conceptions about the subject matter, *e*.*g*., through participation in training by experts or examination of reliable literature [118]. As conceptions on microplastics are mainly media-driven, teachers should regularly survey their students’ and their own conceptions [110]. In general, schools are lagging behind concerning microplastics. Although microplastics can be integrated into some ecological topics, they are not yet a fixed part of the curriculum, leaving a great need for action.

## Conclusions

Microplastics are one of our generation’s critical environmental challenges. A potential pathway for successful reduction of exposure may lie in recognizing and building upon individual conceptions, which often contain alternative (non-scientific) conceptions [64]. Consequently, knowledge about individual conceptions provides valuable starting points for successful awareness campaigns aiming to educate [60]. A key to bridge the “ivory tower” of expert knowledge to the general public is appropriate science communication (jointly initiated by teams of scientists and educators). For reaching this aim, the communication must be tailored to the audience, be understandable and straightforward in content and language, create a connection to the audiences’ lifeworld and take (alternative) conceptions into account [119, 128]. In acknowledging these major domains of science communication, scientists can share their expertise with the public, and educators can support the most promising channels to shape the public’s awareness sustainably. As humans are the cause, they are also the solution [59].

## Limitations

The present study was conducted among students. Within this sample, we aimed to cover different study fields by collecting data at the six different faculties of the University of Bayreuth. However, the sample does not represent the overall German population, wherefore the results cannot be generalized. The study must be read accordingly. The open questions are a suitable and well-established method to collect data on individual conceptions; however, due to the time restrictions and the artificial situation reminiscent of an exam, students may not have written down every experience and idea they hold on the specific topics in connection with microplastics. Finally, the study allowed to capture only selected issues related to microplastics. Further studies are needed to receive a more comprehensive understanding of students’ conceptions of microplastics.
